# Sparse Bayesian Learning for DOA Estimation with Mutual Coupling

**DOI:** 10.3390/s151026267

**Published:** 2015-10-16

**Authors:** Jisheng Dai, Nan Hu, Weichao Xu, Chunqi Chang

**Affiliations:** 1 School of Electrical and Information Engineering, Jiangsu University, 301 Xuefu Road, Zhenjiang 212013, China; 2 National Mobile Communications Research Laboratory, Southeast University, 2 Sipailou Road, Nanjing 210096, China; 3 School of Electronic and Information Engineering, Soochow University, 178 East Ganjiang Road, Suzhou 215006, China; E-Mails: hunan@suda.edu.cn (N.H.); cqchang@suda.edu.cn (C.C.); 4 Department of Automatic Control, Guangdong University of Technology, 100 Huanxi Road, Guangzhou 510006, China; E-Mail: wcxu@gdut.edu.cn

**Keywords:** Sparse Bayesian Learning (SBL), Direction-of-Arrival (DOA), Uniform Linear Array (ULA), mutual coupling

## Abstract

Sparse Bayesian learning (SBL) has given renewed interest to the problem of direction-of-arrival (DOA) estimation. It is generally assumed that the measurement matrix in SBL is precisely known. Unfortunately, this assumption may be invalid in practice due to the imperfect manifold caused by unknown or misspecified mutual coupling. This paper describes a modified SBL method for joint estimation of DOAs and mutual coupling coefficients with uniform linear arrays (ULAs). Unlike the existing method that only uses stationary priors, our new approach utilizes a hierarchical form of the Student *t* prior to enforce the sparsity of the unknown signal more heavily. We also provide a distinct Bayesian inference for the expectation-maximization (EM) algorithm, which can update the mutual coupling coefficients more efficiently. Another difference is that our method uses an additional singular value decomposition (SVD) to reduce the computational complexity of the signal reconstruction process and the sensitivity to the measurement noise.

## 1. Introduction

The problem of estimating the direction-of-arrival (DOA) of multiple narrow-band sources has received considerable attention in many fields, e.g., radar, sonar, radio astronomy and mobile communications [1]. Many high-resolution DOA estimation algorithms have been proposed in the last few decades. Their excellent performance relies crucially on perfect knowledge of the array manifold. In practice, however, the array manifold usually suffers from imperfections, such as unknown mutual coupling between antenna elements, imperfectly-known sensor positions and orientations and gain-phase imbalances. Without array manifold calibration, the performance of DOA estimation may degrade substantially. Hence, it is necessary to calibrate imperfections prior to carrying out DOA estimation.

A large number of calibration methods has been proposed to deal with the imperfect manifold caused by unknown or misspecified mutual coupling [2,3,4,5,6,7,8,9,10,11]. For example, some iterative mutual coupling auto-calibration methods for uniform linear arrays (ULAs) and uniform circular arrays (UCAs) were proposed in [2,3]. However, the high computational complexity required by iterations may be time consuming, and the convergence is not theoretical guaranteed, thus resulting in algorithmic instability. Recently, by taking advantage of the special structure of the mutual coupling matrix for ULAs, Ye and coauthors [4,5] demonstrated that DOAs can be accurately estimated without compensating for mutual coupling if a group of auxiliary sensors are added into ULAs. Dai and coauthors further extended the result to the spatial smoothing method [6], real-valued method [7] and $l_{1}$-norm regularized method [12]. These methods are referred to as the auxiliary methods. Neither a calibration source nor iteration is required in the auxiliary methods. However, because of only applying a middle subarray, they reduce the working array aperture, which limits the applicability of these methods to cases with either few DOAs or many array elements. Some alternatives that can calibrate the imperfect manifold with the whole array in the presence of mutual coupling are proposed in [8,9,10,11].

Recently, the emerging technique of compressive sensing (CS) has given renewed interest to the problem of DOA estimation [12,13,14,15,16,17,18]. These methods exhibit many advantages, e.g., improved robustness to noise, limited number of snapshots and correlation of signals. Sparse Bayesian learning (SBL) is a popular and important technique for the sparse signal recovery in CS [19,20,21], which formulates the signal recovery problem from a Bayesian perspective, while the sparsity information is exploited by assuming a sparse prior for the signal of interest. It is worth noting that $l_{1}$-norm regularized optimization is deemed to be a special example of SBL, if a maximum *a posteriori* (MAP) optimal estimate is adopted with a Laplace signal prior. Theoretical and empirical results show that SBL methods can achieve enhanced performance over $l_{1}$-norm regularized optimization [20,21]. It is generally assumed that the measurement matrix in SBL is precisely known. Unfortunately, this assumption is invalid if perturbations on the measurement matrix (e.g., when the array manifold suffers from the aforementioned imperfections) are considered. Additive perturbations [22,23] and multiplicative perturbations [16] have been addressed in the recent literature.

In this paper, we will propose a modified Bayesian method for joint estimation of DOAs and the mutual coupling coefficients with ULAs, where the optimization problem is formulated in a SBL framework with a two-stage hierarchical prior, and then, we adopt an expectation-maximization (EM) algorithm that treats the signals of interest and the mutual coupling coefficients as hidden variables and parameters, respectively. To the best of our knowledge, there are very limited SBL methods that can resolve the DOAs with mutual coupling. This problem was first addressed in [24], where a unified SBL framework was proposed to address the DOA estimation problem with typical perturbations of mutual coupling, gain-phase uncertainty and sensor location error. The main differences between our method and the method in [24] are:

- Our SBL model constitutes a two-stage hierarchical form, which results in the Student *t* prior in a hierarchical manner; while the model in [24] has only stationary priors. The advantage of the Student *t* prior is that it enforces the sparsity constraint more heavily [21,25].
- Our method provides a distinct Bayesian inference for the EM algorithm, which can update the mutual coupling coefficients more efficiently.
- Our method uses an additional singular value decomposition (SVD) to reduce the computational complexity of the signal reconstruction process and the sensitivity to the measurement noise.

These differences guarantee that our method can give a better joint estimation performance of DOAs and the mutual coupling coefficients.

## 2. Data Model from the Bayesian Viewpoint

### 2.1. DOA Estimation Model

Consider *K* narrow-band far-field sources impinging on an *M*-element ULA, where the distance between adjacent sensors is *d*. The *K* signals, $s_{1}\left(t\right),s_{2}\left(t\right),\ldots ,s_{K}\left(t\right)$, arrive at the array from distinct directions, $\theta_{1},\theta_{2},\ldots ,\theta_{K}$, with respect to the normal line of the array. The M×1 array output vector y(t) is then given by:

(1) $$\begin{array}{c} y\left(t\right)=CAs\left(t\right)+n\left(t\right),\;\;\;t\in \left\{t_{1},t_{2},\ldots ,t_{T}\right\} \end{array}$$

where $y\left(t\right)={\left[y_{1}\left(t\right),y_{2}\left(t\right),\ldots ,y_{M}\left(t\right)\right]}^{T}$, $s\left(t\right)={\left[s_{1}\left(t\right),s_{2}\left(t\right),\ldots ,s_{K}\left(t\right)\right]}^{T}$, $A=[\alpha \left(\theta_{1}\right),\alpha \left(\theta_{2}\right),\ldots ,\alpha \left(\theta_{K}\right)]$, $\alpha \left(\theta_{k}\right)={\left[1,e^{j\phi (\theta_{k})},\ldots ,e^{j\left(M-1\right)\phi \left(\theta_{k}\right)}\right]}^{T}$, $\phi \left(\theta_{k}\right)=\left(-2\pi d/\lambda \right)\sin\left(\theta_{k}\right)$ and $n\left(t\right)={\left[n_{1}\left(t\right),n_{2}\left(t\right),\ldots ,n_{M}\left(t\right)\right]}^{T}$ is an unknown noise vector. The matrix $C\in C^{M\times M}$ is the mutual coupling matrix (MCM) for ULAs. Many theoretical or experimental studies [26,27] have demonstrated that a banded symmetric Toeplitz MCM with *m* ($\leq \frac{M-1}{2}$) coefficients can provide a good approximation of real-world situations, *i.e.*, C=toeplitz(c), where $c=[c_{0},c_{1},c_{2},\ldots ,c_{m}]$ with $0<|c_{1}\left|,\right|c_{2}\left|,\ldots ,\right|c_{m}|<c_{0}=1$ and toeplitz(c) denotes a symmetric Toeplitz matrix constructed by the vector c.

Denoting $Y=[y\left(t_{1}\right),y\left(t_{2}\right),\ldots ,y\left(t_{T}\right)]$, $N=[n\left(t_{1}\right),n\left(t_{2}\right),\ldots ,n\left(t_{T}\right)]$ and $S=[s\left(t_{1}\right),s\left(t_{2}\right),\ldots ,s\left(t_{T}\right)]$, we have:

(2) $$\begin{array}{c} Y=CAS+N. \end{array}$$

Following the convention in [14], we use the singular value decomposition (SVD) to reduce the computational complex of the signal reconstruction process and the sensitivity to the measurement noise. Let the SVD of Y be written in the form of:

(3) $$\begin{array}{c} Y=U_{s}\Lambda_{s}V_{s}^{H}+U_{n}\Lambda_{n}V_{n}^{H} \end{array}$$

where $U_{s}\in C^{M\times \hat{K}}$ and $V_{s}\in C^{T\times \hat{K}}$ are unitary matrices whose columns are the singular vectors corresponding to the $\hat{K}$ largest singular values, while the columns of $U_{n}\in C^{M\times (M-\hat{K})}$ and $V_{n}\in C^{T\times (M-\hat{K})}$ are the singular vectors corresponding to the rest $M-\hat{K}$ singular values. Note that the value of $\hat{K}$ is generally determined by the singular values; especially if the number of sources *K* is exactly known, $\hat{K}$ is set to *K*.

Using Equation (2) and defining $\hat{Y}=YV_{s}$, we obtain:

(4) $$\begin{array}{c} \hat{Y}=CA\hat{S}+\hat{N} \end{array}$$

where $\hat{S}=SV_{s}$ and $\hat{N}=NV_{s}$. In order to cast the problem of DOA estimation with unknown mutual coupling as a sparse representation problem, we let Ω denote the set of possible locations and let $\hat{\theta }$ be a generic location parameter. Furthermore, let ${\left\{{\hat{\theta }}_{i}\right\}}_{i=1}^{K_{\hat{\theta }}}$ denote a grid that covers Ω. If the grid is fine enough, such that the true DOAs lie on (or, practically, close to) the grid, we can use the following model for $\hat{Y}$:

(5) $$\begin{array}{c} \hat{Y}=CA_{\hat{\theta }}{\hat{S}}_{\hat{\theta }}+\hat{N} \end{array}$$

where $A_{\hat{\theta }}≜\left[\alpha \left({\hat{\theta }}_{1}\right),\alpha \left({\hat{\theta }}_{2}\right),\ldots ,\alpha \left({\hat{\theta }}_{K_{\hat{\theta }}}\right)\right]$ and ${\hat{S}}_{\hat{\theta }}$ is a $K_{\hat{\theta }}\times \hat{K}$ complex matrix whose *i*-th row corresponds to the signal impinging on the array from a possible source at ${\hat{\theta }}_{i}$. It is easy to verify that the *i*-th row is nonzero and equals the *k*-th row of $\hat{S}$ if signal *k* comes from ${\hat{\theta }}_{i}$ for some *k* and zero otherwise. As a result, the aim of this paper is to find a row-sparse ${\hat{S}}_{\hat{\theta }}$ (in other words, with a few nonzero rows) and a Toeplitz matrix C that minimize the following objective function:

(6) $$\begin{array}{c} ∥\hat{Y}-CA_{\hat{\theta }}{\hat{S}}_{\hat{\theta }}{∥}_{2} \end{array}$$

where ${∥\cdot ∥}_{2}$ stands for the Frobenius norm. Finding the sparse solution to the above problem through the $l_{1}$-norm regularized optimization is intractable, as it is a non-convex optimization problem with unknown C, which cannot be solved in polynomial time. In the next section, we will propose an SBL method for the DOA estimation in the presence of unknown coupling. To this end, we have to preliminarily model the noisy and sparse signals as in [19].

### 2.2. Noise Model

Firstly, we address the noise model that is commonly used in SBL. Assume elements in the noise vector n defined in Equation (1) are independent and each has a complex Gaussian distribution with zero mean and a common variance $\sigma^{2}$. Since the orthogonal invariance property of the Gaussian random matrix makes the distribution impervious to multiplication by orthogonal matrices, each element in $\hat{N}$ is approximately i.i.d. complex Gaussian with the same mean and variance [15,28], *i.e.*, $p\left({\hat{N}}_{i,j}\right)=\mathrm{CN}\left({\hat{N}}_{i,j}|0,\beta^{-1}\right)$, where $\beta =\sigma^{-2}$ denotes the noise precision and ${\hat{N}}_{i,j}$ is the (i,j)-th element in $\hat{N}$. Then, we have:

(7) $$\begin{array}{c} p\left(\hat{Y}|{\hat{S}}_{\hat{\theta }},\beta ;\;C\right)=\prod_{k=1}^{\hat{K}}\mathrm{CN}\left({\hat{y}}_{k}|CA_{\hat{\theta }}{\hat{s}}_{k},\beta^{-1}I\right) \end{array}$$

where ${\hat{y}}_{k}$ and ${\hat{s}}_{k}$ denote the *k*-th columns of $\hat{Y}$ and ${\hat{S}}_{\hat{\theta }}$, respectively. Usually, the noise variance $\sigma^{2}$ is unknown, so is the noise precision *β*. Hence, we model *β* as a Gamma hyperprior:

(8) $$\begin{array}{c} p(\beta )=\Gamma (\beta ;\;a,b) \end{array}$$

where we set a,b→0 as in [19,21] so as to obtain a broad hyperprior. The reason why we choose the Gamma hyperprior is that it is a conjugate prior (in Bayesian probability theory, a prior p(θ) is said to be conjugate to p(x|θ) if the posterior distribution p(θ|x) has the same functional form as the prior) of the Gaussian distribution.

### 2.3. Sparse Signal Model

A widely-used sparseness prior for ${\hat{S}}_{\hat{\theta }}$ is the Laplace distribution; however, it is not readily accomplished with such a prior in SBL, because the Laplace prior is not conjugate to the Gaussian likelihood, and hence, it is unlikely to perform the associated Bayesian inference in closed form [21]. A typical SBL treatment of ${\hat{S}}_{\hat{\theta }}$, proposed in RVM [19], begins by assigning a non-stationary Gaussian prior distribution with a distinct inverse variance $\delta_{i}$ for each row of ${\hat{S}}_{\hat{\theta }}$. Letting $\delta ={\left[\delta_{1},\delta_{2},\ldots ,\delta_{K_{\hat{\theta }}}\right]}^{T}$ and Δ=diag(δ), we have:

(9) $$\begin{array}{c} p\left({\hat{S}}_{\hat{\theta }}|\delta \right)=\prod_{k=1}^{\hat{K}}\mathrm{CN}\left({\hat{s}}_{k}|0,\Delta^{-1}\right) \end{array}$$

In order to make the Bayesian inference convenient and to obtain a two-stage hierarchical prior that favors most rows of ${\hat{S}}_{\hat{\theta }}$ being zeros, the hyper-parameter $\delta_{i}$’s are further modeled as independent Gamma distributions [19,21], *i.e.*,

(10) $$\begin{array}{c} p\left(\delta \right)=\prod_{i=1}^{K_{\hat{\theta }}}\Gamma \left(\delta_{i};\;c,d\right) \end{array}$$

where c→1 and d→0. In this case, the integral $\int_{0}^{\infty }\mathrm{CN}\left({\hat{s}}_{k}|0,\Delta^{-1}\right)\cdot p\left(\delta ;\;c,d\right)d\delta$ corresponds to the Student *t* distribution [19], which can be evaluated analytically. Alternatively, a two-stage hierarchical method that results in Laplace priors was addressed in [21].

## 3. The Proposed Sparse Bayesian Learning Method

The associated learning problem becomes the search for the hyperparameters *β* and ***δ***, as well as the parameter C. As $p({\hat{S}}_{\hat{\theta }},\beta ,\delta |\hat{Y};\;C)$ cannot be explicitly calculated, a Type-II ML [19] (or evidence maximization) procedure is exploited to perform the Bayesian inference. In other words, the hyperparameters (*β* and ***δ***) and parameter (C) are estimated by maximizing $p(\hat{Y}|\beta ,\delta ;\;C)$ or its logarithm $L\left(\beta ,\delta ;\;C\right)≜\ln p\left(\hat{Y}|\beta ,\delta ;\;C\right)$. However, explicitly finding the values of *β*, ***δ*** and C that maximize L(β,δ;C) is intractable, and here, we adopt an EM algorithm that treats ${\hat{S}}_{\hat{\theta }}$ as a hidden variable. The principle behind the EM algorithm is to instead repeatedly construct a lower bound on L(β,δ;C) (E-step) and then to optimize that lower-bound (M-step). Specifically,

- E-step: Compute:
  (11) $$p({\hat{S}}_{\hat{\theta }}|\hat{Y},\beta ,\delta ;\;C)$$
  and evaluate:
  (12) $${\langle \ln\;p(\hat{Y},{\hat{S}}_{\hat{\theta }},\beta ,\delta ;\;C)\rangle }_{E^{\left(t\right)}}≜E_{p({\hat{S}}_{\hat{\theta }}|\hat{Y},\beta^{\left(t\right)},\delta^{\left(t\right)};\;C^{\left(t\right)})}\left[\ln\;p(\hat{Y},{\hat{S}}_{\hat{\theta }},\beta ,\delta ;\;C)\right]$$
  where ${\left(\cdot \right)}^{\left(t\right)}$ denotes the estimated value in the *t*-th iteration.
- M-step: Find the hyperparameter updates and the parameter updates:
  (13) $$\begin{array}{c} \left\{\beta^{\left(t+1\right)},\delta^{\left(t+1\right)},C^{\left(t+1\right)}\right\}=\arg\max_{\beta ,\delta ,C}\;{\langle \ln\;p(\hat{Y},{\hat{S}}_{\hat{\theta }},\beta ,\delta ;\;C)\rangle }_{E^{\left(t\right)}}. \end{array}$$

In the rest of this section, we will discuss the two steps in detail.

### 3.1. E-Step

Using the Bayes rule, we can verify that the posterior distribution of ${\hat{S}}_{\hat{\theta }}$ in Equation (11) is also a complex Gaussian [19]:

(14) $$p({\hat{S}}_{\hat{\theta }}|\hat{Y},\beta ,\delta ;\;C)=\frac{p\left(\hat{Y}|{\hat{S}}_{\hat{\theta }},\beta ;C\right)\cdot p\left({\hat{S}}_{\hat{\theta }}|\delta \right)}{p(\hat{Y}|\beta ,\delta ;\;C)}=\prod_{k=1}^{\hat{K}}\mathrm{CN}\left({\hat{s}}_{k}|\mu_{k},\Sigma \right)$$

where:

(15) $$\mu_{k}=\beta \Sigma A_{\hat{\theta }}^{H}C^{H}{\hat{y}}_{k},\;\;\;k=1,2,\ldots ,\hat{K}$$

(16) $$\Sigma ={\left(\beta A_{\hat{\theta }}^{H}C^{H}CA_{\hat{\theta }}+\Delta \right)}^{-1}=\Delta^{-1}-\Delta^{-1}{\left(CA_{\hat{\theta }}\right)}^{H}{\left(\beta^{-1}I+CA_{\hat{\theta }}\Delta^{-1}{\left(CA_{\hat{\theta }}\right)}^{H}\right)}^{-1}CA_{\hat{\theta }}\Delta^{-1}.$$

On the other hand, by combining the stages of the hierarchical Bayesian model, $p(\hat{Y},{\hat{S}}_{\hat{\theta }},\beta ,\delta ;\;C)$ can be rewritten as:

(17) $$p\left(\hat{Y},{\hat{S}}_{\hat{\theta }},\beta ,\delta ;\;C\right)=p\left(\hat{Y}|{\hat{S}}_{\hat{\theta }},\beta ;\;C\right)p\left({\hat{S}}_{\hat{\theta }}|\delta \right)p\left(\beta \right)p\left(\delta \right).$$

Therefore, Equation (12) leads to:

(18) $${\langle \ln\;p(\hat{Y},{\hat{S}}_{\hat{\theta }},\beta ,\delta ;\;C)\rangle }_{E^{\left(t\right)}}={\langle \ln\;p\left(\hat{Y}|{\hat{S}}_{\hat{\theta }},\beta ;\;C\right)p\left({\hat{S}}_{\hat{\theta }}|\delta \right)p\left(\beta \right)p\left(\delta \right)\rangle }_{E^{\left(t\right)}}.$$

### 3.2. M-Step

In this subsection, we will address the derivation of hyperparameter updates ($\beta^{\left(t+1\right)}$ and $\delta^{\left(t+1\right)}$), as well as the parameter update ($C^{\left(t+1\right)}$).

1. For *β*, ignoring terms in the logarithm independent thereof, we just have to maximize:
  $$\begin{array}{cc} & {\langle \ln\;p\left(\hat{Y}|{\hat{S}}_{\hat{\theta }},\beta ;\;C\right)p\left(\beta \right)\rangle }_{E^{\left(t\right)}} \end{array}$$
  $$\begin{array}{cc} = & {\langle \ln\;p(\hat{Y}|{\hat{S}}_{\hat{\theta }},\beta ;\;C)\rangle }_{E^{\left(t\right)}}+\ln\;p\left(\beta \right) \end{array}$$
  $$\begin{array}{cc} = & \hat{K}M\ln\beta -\beta \sum_{k=1}^{\hat{K}}{\langle ∥{\hat{y}}_{k}-CA_{\hat{\theta }}{\hat{s}}_{k}{∥}_{2}^{2}\rangle }_{E^{\left(t\right)}}+\left(a-1\right)\ln\beta -b\beta +\mathrm{const} \end{array}$$
  $$\begin{array}{cc} = & \hat{K}M\ln\beta -\beta \sum_{k=1}^{\hat{K}}{∥{\hat{y}}_{k}-C^{\left(t\right)}A_{\hat{\theta }}\mu_{k}^{\left(t\right)}∥}_{2}^{2} \end{array}$$
  (19) $$\begin{array}{cc} & \;\;\;\;\;\;\;\;\;\;\;\;-\beta \hat{K}\mathrm{tr}\left(C^{\left(t\right)}A_{\hat{\theta }}\Sigma^{\left(t\right)}{\left(C^{\left(t\right)}A_{\hat{\theta }}\right)}^{H}\right)+\left(a-1\right)\ln\beta -b\beta +\mathrm{const} \end{array}$$
  which, through differentiation, gives the update for $\beta^{\left(t+1\right)}$:
  (20) $$\beta^{\left(t+1\right)}=\frac{\hat{K}M+\left(a-1\right)}{\left(\begin{array}{c} b+\sum_{k=1}^{\hat{K}}{∥{\hat{y}}_{k}-C^{\left(t\right)}A_{\hat{\theta }}\mu_{k}^{\left(t\right)}∥}_{2}^{2}\\ \;+\hat{K}\mathrm{tr}\left(C^{\left(t\right)}A_{\hat{\theta }}\Sigma^{\left(t\right)}{\left(C^{\left(t\right)}A_{\hat{\theta }}\right)}^{H}\right) \end{array}\right)}.$$
2. For ***δ***, also ignoring terms in the logarithm independent thereof, we have:
  $$\begin{array}{cc} & {\langle \ln\;p\left({\hat{S}}_{\hat{\theta }}|\delta \right)p\left(\delta \right)\rangle }_{E^{\left(t\right)}} \end{array}$$
  $$\begin{array}{cc} = & {\langle \ln\;p({\hat{S}}_{\hat{\theta }}|\delta )\rangle }_{E^{\left(t\right)}}+\ln\;p\left(\delta \right) \end{array}$$
  $$\begin{array}{cc} = & \hat{K}\ln\left|\Delta \right|-\sum_{k=1}^{\hat{K}}{\langle {\hat{s}}_{k}^{H}\Delta {\hat{s}}_{k}\rangle }_{E^{\left(t\right)}}-d\sum_{i=1}^{K_{\hat{\theta }}}\delta_{i}+\left(c-1\right)\sum_{i=1}^{K_{\hat{\theta }}}\ln\delta_{i}+\mathrm{const} \end{array}$$
  (21) $$\begin{array}{cc} = & \left(\hat{K}+c-1\right)\sum_{i=1}^{K_{\hat{\theta }}}\ln\delta_{i}-d\sum_{i}^{K_{\hat{\theta }}}\delta_{i}-\sum_{k=1}^{\hat{K}}\mathrm{tr}\left(\left(\mu_{k}^{\left(t\right)}{\left(\mu_{k}^{\left(t\right)}\right)}^{H}+\Sigma^{\left(t\right)}\right)\Delta \right)+\mathrm{const}. \end{array}$$
  Setting the derivative of Equation (21), with respect to ***δ***, to zero and solving for each $\delta_{i}$ gives the updates:
  (22) $$\begin{array}{c} \delta_{i}^{\left(t+1\right)}=\frac{\hat{K}+c-1}{d+\sum_{k=1}^{\hat{K}}{\left[\Xi_{k}^{\left(t\right)}\right]}_{ii}},\;\;\;i=1,2,\ldots ,K_{\hat{\theta }} \end{array}$$
  where $\Xi_{k}^{\left(t\right)}≜\mu_{k}^{\left(t\right)}{\left(\mu_{k}^{\left(t\right)}\right)}^{H}+\Sigma^{\left(t\right)}$.
3. For C, its estimate should maximize the following expected value:
  (23) $$\begin{array}{cc} & {\langle \ln\;p(\hat{Y}|{\hat{S}}_{\hat{\theta }},\beta ;\;C)\rangle }_{E^{\left(t\right)}}=-\beta \sum_{k=1}^{\hat{K}}{∥{\hat{y}}_{k}-CA_{\hat{\theta }}\mu_{k}^{\left(t\right)}∥}_{2}^{2}-\beta \hat{K}\mathrm{tr}\left(CA_{\hat{\theta }}\Sigma^{\left(t\right)}{\left(CA_{\hat{\theta }}\right)}^{H}\right)+\mathrm{const}. \end{array}$$
  In order to calculate the derivative of Equation (23), we need the following lemma.

**Lemma 1** (see [2]). *Let* C *and* c *be defined as earlier, then for any vector* x, *we have:*

(24) $$\begin{array}{c} Cx=T(x)c \end{array}$$

*where the matrix* $T\left(x\right)\in C^{M\times (m+1)}$ *is the sum of the two* M×(m+1) *matrices:*

(25) $$\begin{array}{c} {\left[T_{1}\left(x\right)\right]}_{p,q}=\left\{\begin{array}{cc} {\left[x\right]}_{p+q-1} & p+q\leq M+1\\ 0 & otherwise \end{array}\right. \end{array}$$

(26) $$\begin{array}{c} {\left[T_{2}\left(x\right)\right]}_{p,q}=\left\{\begin{array}{cc} {\left[x\right]}_{p-q+1} & p\geq q\geq 2\\ 0 & otherwise \end{array}\right. \end{array}$$

With the assistance of Lemma 1, we are capable of rewriting the terms $∥{\hat{y}}_{k}-CA_{\hat{\theta }}\mu_{k}^{\left(t\right)}{∥}_{2}^{2}$ and $\mathrm{tr}\left(CA_{\hat{\theta }}\Sigma^{\left(t\right)}{\left(CA_{\hat{\theta }}\right)}^{H}\right)$ in Equation (23) as:

(27) $$\begin{array}{c} ∥{\hat{y}}_{k}-CA_{\hat{\theta }}\mu_{k}^{\left(t\right)}{∥}_{2}^{2}={∥{\hat{y}}_{k}-T\left(A_{\hat{\theta }}\mu_{k}^{\left(t\right)}\right)c∥}_{2}^{2} \end{array}$$

and:

(28) $$\begin{array}{c} \mathrm{tr}\left(CA_{\hat{\theta }}\Sigma^{\left(t\right)}{\left(CA_{\hat{\theta }}\right)}^{H}\right)=∥CD^{\left(t\right)}{∥}_{2}^{2}=\sum_{i=1}^{M}∥Cd_{i}^{\left(t\right)}{∥}_{2}^{2}=\sum_{i=1}^{M}{∥T\left(d_{i}^{\left(t\right)}\right)c∥}_{2}^{2} \end{array}$$

respectively, where $D^{\left(t\right)}{\left(D^{\left(t\right)}\right)}^{H}$ stands for a decomposition of $A_{\hat{\theta }}\Sigma^{\left(t\right)}A_{\hat{\theta }}^{H}$ and $d_{i}^{\left(t\right)}$ denotes the *i*-th column of $D^{\left(t\right)}$. Using Equations (27) and (28), we can calculate the derivative of ${\langle \ln\;p(\hat{Y}|{\hat{S}}_{\hat{\theta }},\beta ;\;C)\rangle }_{p^{\left(t\right)}}$, with respect to c, as:

$$\begin{array}{cc} & \frac{\partial }{\partial c}{\langle \ln\;p(\hat{Y}|{\hat{S}}_{\hat{\theta }},\beta ;\;C)\rangle }_{p^{\left(t\right)}} \end{array}$$

(29) $$\begin{array}{cc} = & \beta \sum_{k=1}^{\hat{K}}T{\left(A_{\hat{\theta }}\mu_{k}^{\left(t\right)}\right)}^{H}\left({\hat{y}}_{k}-T\left(A_{\hat{\theta }}\mu_{k}^{\left(t\right)}\right)c\right)-\beta \hat{K}\sum_{i=1}^{M}T{\left(d_{i}^{\left(t\right)}\right)}^{H}T\left(d_{i}^{\left(t\right)}\right)c. \end{array}$$

Setting the derivative to zero gives:

(30) $$\begin{array}{cc} c= & \left(\sum_{k=1}^{\hat{K}}T{\left(A_{\hat{\theta }}\mu_{k}^{\left(t\right)}\right)}^{H}T\left(A_{\hat{\theta }}\mu_{k}^{\left(t\right)}\right)\right.{\left.+\hat{K}\sum_{i=1}^{M}T{\left(d_{i}^{\left(t\right)}\right)}^{H}T\left(d_{i}^{\left(t\right)}\right)\right)}^{-1}\cdot \sum_{k=1}^{\hat{K}}T{\left(A_{\hat{\theta }}\mu_{k}^{\left(t\right)}\right)}^{H}{\hat{y}}_{k}. \end{array}$$

**Remark 1.** *Note that [9,10,11] provided an alternative parameterization for the steering vector:*

(31) $$\begin{array}{c} C\alpha \left(\theta_{k}\right)=g\left(\theta_{k},c\right)\bar{T}\left(\theta_{k}\right)\tau \left(\theta_{k},c\right) \end{array}$$

*where* $g\left(\theta_{k},c\right)≜\sum_{m^{\prime }=-m}^{m}c_{|m^{\prime }|}e^{jm^{\prime }\phi \left(\theta_{k}\right)}$, $\bar{T}\left(\theta_{k}\right)≜\mathrm{blkdiag}\left\{{\bar{T}}_{1}\left(\theta_{k}\right),{\bar{T}}_{2}\left(\theta_{k}\right),{\bar{T}}_{3}\left(\theta_{k}\right)\right\}$ *is a block diagonal matrix with* ${\bar{T}}_{1}\left(\theta_{k}\right)=\mathrm{diag}\left\{1,e^{j\phi (\theta_{k})},\ldots ,e^{j\left(m-1\right)\phi \left(\theta_{k}\right)}\right\}$, ${\bar{T}}_{2}\left(\theta_{k}\right)={\left[e^{jm\phi (\theta_{k})},\ldots ,e^{j\left(M-m-1\right)\phi \left(\theta_{k}\right)}\right]}^{T}$ *and* ${\bar{T}}_{3}\left(\theta_{k}\right)=\mathrm{diag}\left\{e^{j\left(M-m\right)\phi \left(\theta_{k}\right)},\ldots ,e^{j\left(M-1\right)\phi \left(\theta_{k}\right)}\right\}$ *and* $\tau \left(\theta_{k},c\right)≜\left[\nu_{1},\ldots ,\nu_{m},1,\eta_{1},\ldots ,\eta_{m}\right]$ *with* $\nu_{k}=\frac{1}{g(\theta_{k},c)}\left(\sum_{m^{\prime }=1-k}^{m}c_{|m^{\prime }|}e^{jm^{\prime }\phi \left(\theta_{k}\right)}\right)$ *and* $\eta_{k}=\frac{1}{g(\theta_{k},c)}\left(\sum_{m^{\prime }=-m}^{m-k}c_{|m^{\prime }|}e^{jm^{\prime }\phi \left(\theta_{k}\right)}\right)$. *However, it is unlikely that Equation (31) can be applied to the M-step for* C. *The most difficult aspect for applying the alternative parameterization in Equation (31) is that it is dependent on the geometric progression in* $\alpha (\theta_{k})$; *while the terms* $A_{\hat{\theta }}\mu_{k}^{\left(t\right)}$ *and* $d_{i}^{\left(t\right)}$ *in Equations (27) and (28) do not have the property of geometric progression.*

The EM algorithm proceeds by repeated application of Equations (20), (22) and (30), until a prescribed accuracy is achieved. Once the algorithm is convergent, we are able to obtain the calibrated C. As far as the estimated $\delta_{i}$’s are concerned, we observed that many of $\delta_{i}$ tend to infinity in the EM process. Clearly, the rows corresponding to these infinity $\delta_{i}$’s are zero; while the rows corresponding to small $\delta_{i}$’s imply the true DOAs.

## 4. Simulation Results

In this section, we will present several simulation results to illustrate the performance of our proposed method. We will compare the proposed method to the original SBL method in [24], the iterative method in [3] and the auxiliary methods in [5,12].

**Figure 1:**
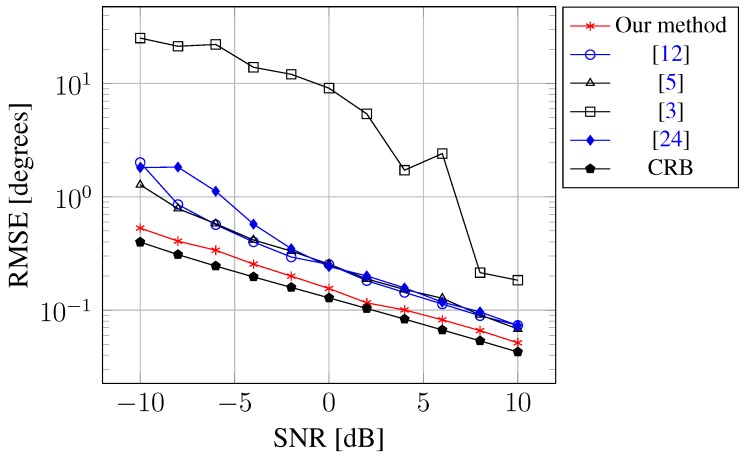
RMSE of the DOA estimate against SNR.

Simulation 1 addresses the performance comparisons of the joint estimation of DOAs and mutual coupling coefficients. Consider a scenario where a ULA composed of M=10 sensors is used to receive K=2 uncorrelated signals coming from $\theta_{1}$ = –19.7 °C and $\theta_{2}$ = 10.1 °C. Note that the effect of mutual coupling is negligible between two sensors that are far enough away from each other, because the mutual coupling coefficient is inversely proportional to their distance. Hence, it is reasonable to approximate the mutual coupling effect with just a few nonzero coefficients. In the simulation, we assume that the number of mutual coupling coefficients is m=2 with $c_{1}=0.5-0.4i$ and $c_{2}=0.3+0.1i$. Figure 1 shows the root mean square error (RMSE) of DOA estimation *versus* input SNR computed via 200 Monte Carlo runs, where the number of snapshots is 100. For the ease of comparison, we also include the curve for CRB. As can be seen from the figure, our method outperforms the state-of-the-art methods. This is because: (1) compared to the method in [24], our method utilizes a hierarchical form of the Student *t* prior to enforce the sparsity of unknown signal more heavily; moreover, our method uses the SVD to reduce the sensitivity to the noise; (2) compared to the methods in [5,12], our method uses the whole array, rather than a subarray, to estimate DOAs and to compensate for the mutual coupling effect.

**Figure 2:**
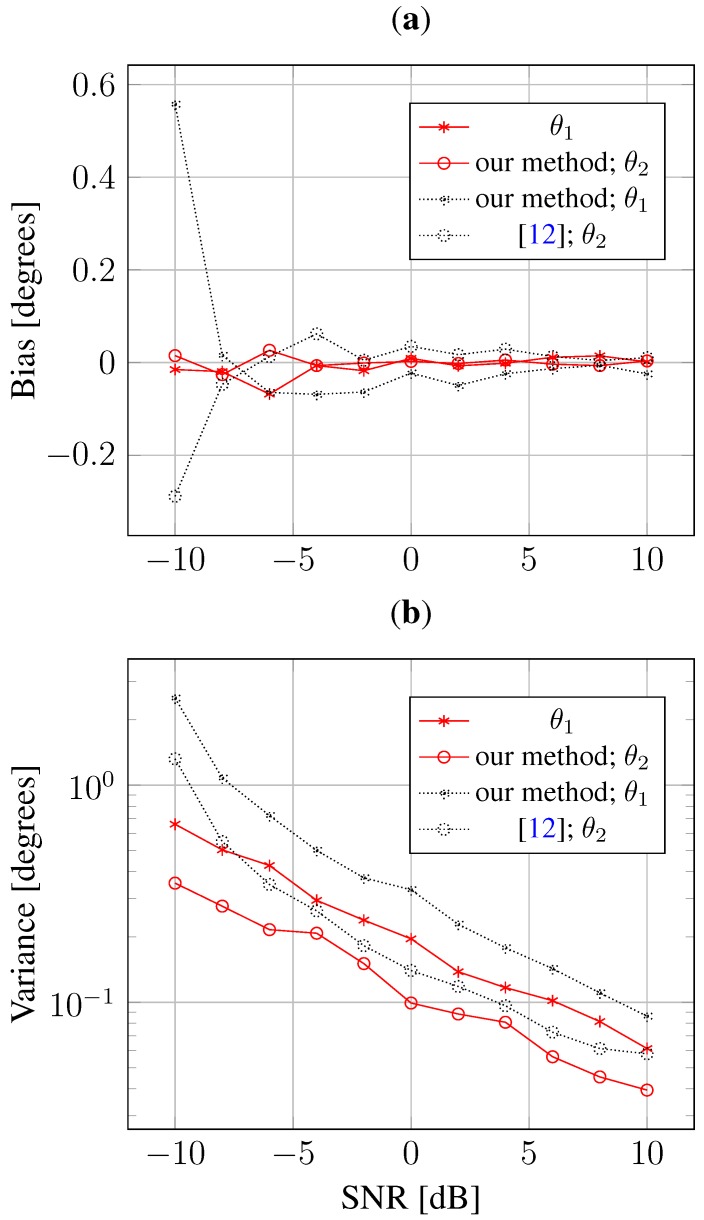
Estimation bias and variance against SNR. (**a**) Bias; (**b**) Variance.

**Figure 3:**
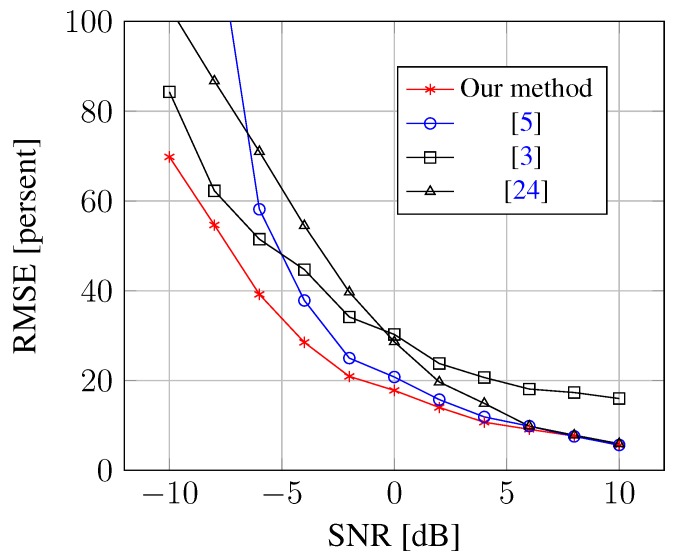
RMSE of mutual coupling coefficients against SNR.

Figure 2 shows the estimation bias and variance for each DOA. Compared to the $l_{1}$-norm regularized method [12], our method can significantly reduce the DOA estimation bias, as well as the variance. To assess the performance of mutual coupling calibration, Figure 3 shows the relative RMSE of the estimation of the mutual coupling coefficients. This relative RMSE is defined as:

(32) $$\begin{array}{c} \sqrt{\frac{1}{200}\sum_{n=1}^{200}\sum_{i=1}^{m}\frac{|c_{ni}-c_{i}{|}^{2}}{|c_{i}{|}^{2}}}\times 100\% \end{array}$$

where $c_{ni}$ is the estimate of $c_{i}$ at the *n*-th Monte Carlo run. As shown from the Figure 3, the mutual coupling coefficients can be estimated more accurately in our method, especially for low SNR signals. Hence, if all of the methods embed the mutual coupling coefficients within the same classical eigendecomposition algorithm, such as MUSIC and ESPRIT, to estimate the DOA, our method can achieve the best performance.

**Figure 4:**
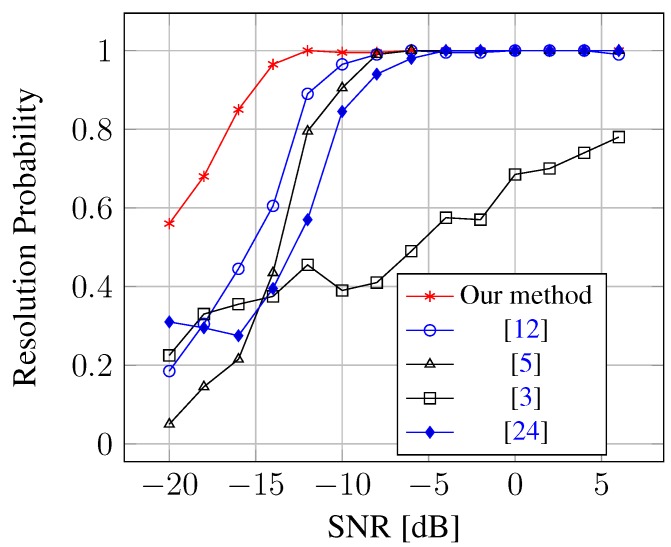
Resolution probability against SNR for closely-spaced sources.

Simulation 2 is used to investigate the capability of resolving closely-spaced sources with limited snapshots (T=50) for several methods. The simulation considers a scenario where a ULA composed of M=13 sensors is used to receive K=2 uncorrelated signals coming from $\theta_{1}$ = –2.5 °C and $\theta_{2}$ = 3.5 °C, and the number of mutual coupling coefficients is m=2 with $c_{1}=0.5-0.4i$ and $c_{2}=0.3+0.1i$. We say that the two signals are exactly resolved in a given run, if $\max_{k=1,2}\left\{\right|{\tilde{\theta }}_{k}-\theta_{k}\left|\right\}$ is smaller than $|\theta_{1}-\theta_{2}|/2$, where ${\tilde{\theta }}_{k}$ stands for the estimated DOA for the *k*-th signal. As can be seen from Figure 4, the resolution performance of our method outperforms others. The superior resolution performance of sparse representation is natural, which is consistent with the simulation results in [12].

**Figure 5:**
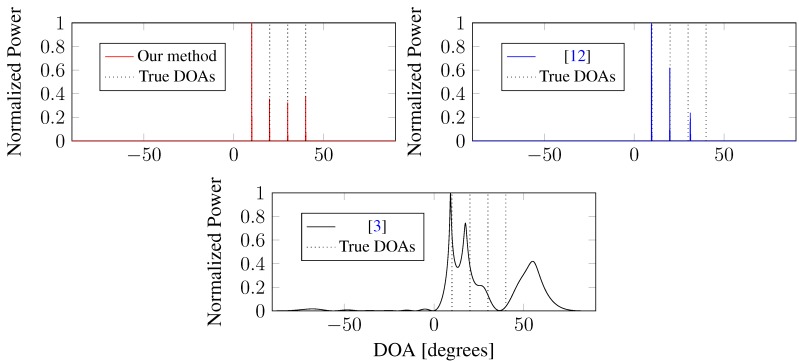
DOA estimation in the case of a “blind angle”.

Simulation 3 addresses the “blind angle” phenomenon, which may result in the substantially degraded performance of DOA estimation [4,5]. We consider a scenario where a ULA composed of M=12 sensors is used to receive K=4 uncorrelated signals coming from $\theta_{1}$ = 10 °C, $\theta_{2}$ = 20 °C, $\theta_{3}$ = 30 °C and $\theta_{4}$ = 40 °C, and the number of mutual coupling coefficients is m=2 with $c_{1}=0.6+0.5i$ and $c_{2}=0.3844-0.3476i$. It is easy to verify that the blind angle occurs at $\theta_{4}$ = 40 °C. The number of snapshots is 30, and the SNR is 10 dB. Figure 5 illustrates that: (1) our method can estimate all of the true DOAs accurately, including the blind DOA $\theta_{4}$ = 40 °C; (2) the $l_{1}$-norm regularized method [12] fails in estimating the blind DOA from $\theta_{4}$ = 40 °C, but succeeds in obtaining the DOAs from other directions; (3) the iterative method [3] misses two DOAs $\theta_{3}$ = 30 °C and $\theta_{4}$ = 40 °C. Obviously, our method has the best performance of “blind angle” suppression.

## 5. Conclusions

We have proposed a modified SBL method that approaches the problem of DOA estimation with unknown mutual coupling. Unlike the original SBL method in [24] that only uses stationary priors, our new method utilized a hierarchical form of the Student *t* prior to enforce the sparsity of unknown signal more heavily. To efficiently perform the Bayesian inference, we adopted a refined EM algorithm that treats ${\hat{S}}_{\hat{\theta }}$ and C as a hidden variable and a parameter, respectively. It can update the mutual coupling coefficients more efficiently. Another difference is that our method used an additional SVD to reduce the computational complexity of the signal reconstruction process and the sensitivity to the measurement noise. Hence, the proposed method is expected to give a better estimation performance. Simulation results have verified its efficiency.
